# Virtual care use prior to emergency department admissions during a stable COVID-19 period in Ontario, Canada

**DOI:** 10.1371/journal.pone.0277065

**Published:** 2023-04-28

**Authors:** Vess Stamenova, Cherry Chu, Emily Borgundvaag, Cathleen Fleury, Janette Brual, Onil Bhattacharyya, Mina Tadrous

**Affiliations:** 1 Women’s College Hospital Institute for Health Systems Solutions and Virtual Care, Women’s College Hospital, Toronto, Ontario, Canada; 2 ICES, Toronto, Ontario, Canada; 3 Department of Family & Community Medicine, University of Toronto, Toronto, Ontario, Canada; 4 Leslie Dan Faculty of Pharmacy, University of Toronto, Toronto, Ontario, Canada; University of Bedfordshire, UNITED KINGDOM

## Abstract

**Background:**

The increased use of telemedicine to provide virtual outpatient visits during the pandemic has led to concerns about potential increased emergency department (ED) admissions and outpatient service use prior to such admissions. We examined the frequency of virtual visits use prior to ED admissions and characterized the patients with prior virtual visit use and the physicians who provided these outpatient visits.

**Methods:**

We conducted a retrospective, population-based, cross-sectional analysis using linked health administrative data in Ontario, Canada to identify patients who had an ED admission between July 1 and September 30, 2021 and patients with an ED admissions during the same period in 2019. We grouped patients based on their use of outpatient services in the 7 days prior to admission and reported their sociodemographic characteristics and healthcare utilization.

**Results:**

There were 1,080,334 ED admissions in 2021 vs. 1,113,230 in 2019. In 2021, 74% of these admissions had no prior outpatient visits (virtual or in-person) within 7 days of admission, compared to 75% in 2019. Only 3% of ED admissions had both virtual and in-person visits in the 7 days prior to ED admission. Patients with prior virtual care use were more likely to be hospitalized than those without any outpatient care (13% vs 7.7.%).

**Interpretation:**

The net amount of ED admissions and outpatient care prior to admission remained the same over a period of the COVID-19 pandemic when cases were relatively stable. Virtual care seemed to be able to appropriately triage patients to the ED and virtual visits replaced in-person visits ahead of ED admissions, as opposed to being additive.

## Introduction

The COVID-19 pandemic has led to the emergence of standard use of virtual visits in health care across the globe [1, 2]. In Ontario, Canada the proportion of ambulatory visits completed virtually has been maintained at slightly above 50% from 2020 to 2021 [3]. Despite its widespread adoption, it is still unclear when virtual visits are clinically appropriate and how such wide use of virtual visits impacts patient outcomes and healthcare utilization metrics.

Before the pandemic, there had been concerns that virtual visits may lead to an increased use of outpatient services with patients having both a virtual and an in-person visit for the same clinical issue in situations where the virtual visit was not sufficient [4, 5]. For example, pre-pandemic data (2007–2016) from Manitoba showed that virtual visit users had on average 1.3 times more ambulatory visits than non-users [6]. In addition, studies have produced mixed evidence with regard to the effect of virtual visits on urgent services such as emergency department (ED) admissions and hospitalizations [7]. Many of the studies reported in the literature are based on data from site-specific programs and therefore have limited generalizability. Finally, policymakers and some physicians have become concerned that the high rates of virtual visits during COVID-19 have led to an increase in emergency department admissions because of poor access to in-person outpatient care [8]. This concern is exacerbated when one considers rural and lower socioeconomic status patients who already had poor access to care before the pandemic [9]. Combined with reports of lower uptake of virtual visits among these patients [10, 11], it is not clear how the transition of care from in-person to virtual impacts ED use.

The high adoption of virtual visits during the pandemic, in the context of a publicly funded healthcare system allowing us access to most visits across the entire population, offers a unique opportunity to examine the frequency of virtual visits use prior to ED admissions. Therefore, the goal of this study was to characterize the frequency and modality (in-person vs virtual) of outpatient care prior to ED admissions. If virtual care replaces in-person care, then we should expect that the proportion of people with both in-person and virtual care before ED admissions should be high relative to those with either modality alone. We, therefore, examined whether there was an overall increase in outpatient visits prior to ED admissions during a period of the pandemic when access to virtual visits was available compared to a seasonality matched period before the pandemic where access to virtual visits was quite limited.

We also aimed to characterize the patients who had a virtual visit prior to an ED admission vs. those who had an in-person visit and the physicians who saw patients with virtual only visits prior to their ED admission compared to those who saw patients virtually or in-person prior to their ED admission.

## Materials and methods

### Study design and population

We conducted a retrospective, population-based, cross-sectional analysis using linked health administrative data in Ontario, Canada to identify all patients who had an ED admission between July 1 and September 30, 2021 and those with ED admissions between July 1 and September 30, 2019. The 2021 summer window represented a relatively stable period of COVID-19, in between the major waves of COVID-19 infection and the 2019 period served as a control period during which access to virtual visits was relatively limited. We excluded patients who had invalid identification numbers, were non-Ontario residents, had ED admissions that were not publicly insured, and those who had another ED admission within 7 days prior to July 1 of the year of interest (full exclusion list is provided in the S1 Appendix).

#### Data sources

Ontario is the province with the largest population in Canada and all permanent residents have public insurance fully covering all necessary physician and hospital services. We used the Ontario Health Insurance Plan (OHIP) for physician claims, the Canadian Institutes of Health Information Discharge Abstract Database (CIHI-DAD) for information about all hospitalizations, the CIHI National Ambulatory Care Reporting System (NACRS) for hospital- and community-based ambulatory care including ED admissions, and the ICES Physician Database (IPDB) for data on physician specialty. Databases were linked using unique identifiers and analyzed at ICES (formerly the Institute for Clinical Evaluative Sciences). Virtual visits were identified as any OHIP claim with the location recorded as “P” for phone, indicating virtual visit services. We then described patients based on characteristics, such as age, sex, chronic disease diagnoses, income quintile (based on postal code), urban vs. rural (based on postal code’s rurality index for Ontario (RIO) where a score below 40 was categorized as urban), Ontario Marginalization Index (ONMARG) containing data on patient economic, ethno-racial, age-based, and social deprivation (details on databases used in the S1 Appendix).

#### Patient groups

Patients were categorized into subgroups based on the type of outpatient visit (in-person vs virtual) they had prior to their ED admission (if any). For patients who had virtual visits prior to their ED admission (within 7 days), subgroups of patients were also created based on whether their last virtual outpatient visit was within 24, 48, 72 hours, or 7 days of their ED admission. The following non-mutually exclusive groups of patients were created:

1. all patients with an ED admission during the study window
2. patients with an ED admission who did not have any virtual visits within the 7 days prior (includes patients who had zero visits or only in-person visits prior to ED admission)
3. patients with an ED admission who did not have any outpatient visits (virtual or in-person) within the 7 days prior
4. patients who had at least one virtual visit within 24 hours prior to an ED admission
5. patients who had at least one virtual visit within 48 hours prior to an ED admission
6. patients who had at least one virtual visit within 72 hours prior to an ED admission
7. patients who had at least one virtual visit within 7 days prior to an ED admission
8. patients who had only in-person visits during the 7 days prior to admission.

Patients may have belonged to more than one subgroup, i.e. the 48 hours virtual care group includes patients in the 24 hours group. All analyses were conducted using SAS version 9.4.

#### Patient and physician characteristics

To compare the characteristics of patients who had a virtual vs. an in-person visit prior to ED admission, we looked at the most recent visit that occurred within 7 days prior to their ED admission. We identified patient characteristics such as age, sex, region of residence, rurality, neighborhood income, marginalization index, chronic conditions, number of ED admissions, hospitalizations and outpatient visits in the year prior to ED admission, number of outpatient visits in the 7 days prior to ED admission, days between outpatient visit and emergency visit, whether the visit was on the same day as the ED admission, and if the ED admission resulted in hospitalization.

To examine characteristics of the outpatient visits that occurred within 7 days before ED admissions, we classified patients into 4 groups. Three of these groups included patients who had visited the ED in 2021: those with any visit (virtual or in-person), those with a virtual visit, and those with an in-person visit prior to ED admission. The last group consisted of patients who had an in-person visit prior to ED admission in 2019 (we did not include a virtual visit group as virtual visits were uncommon in 2019).

We also looked at the characteristics of physicians who had provided the most recent outpatient visit prior to the patient’s ED admission in 2021 and categorized them into two groups based on the type of visit: physicians who had seen patients with only a virtual visit in 2021; physicians who had seen patients with either a virtual or in-person visit in 2021. For physician characteristics, we looked at age, sex, region of and years in practice, and patient daily volume.

See S1 Appendix for detailed definitions.

### Ethics

Use of these databases for the purposes of this study was authorized under §45 of Ontario’s Personal Health Information Protection Act, which does not require review by a research ethics board (REB). All data was de-identified at ICES and individual patient consent was waived.

## Results and discussion

**Fig 1:**
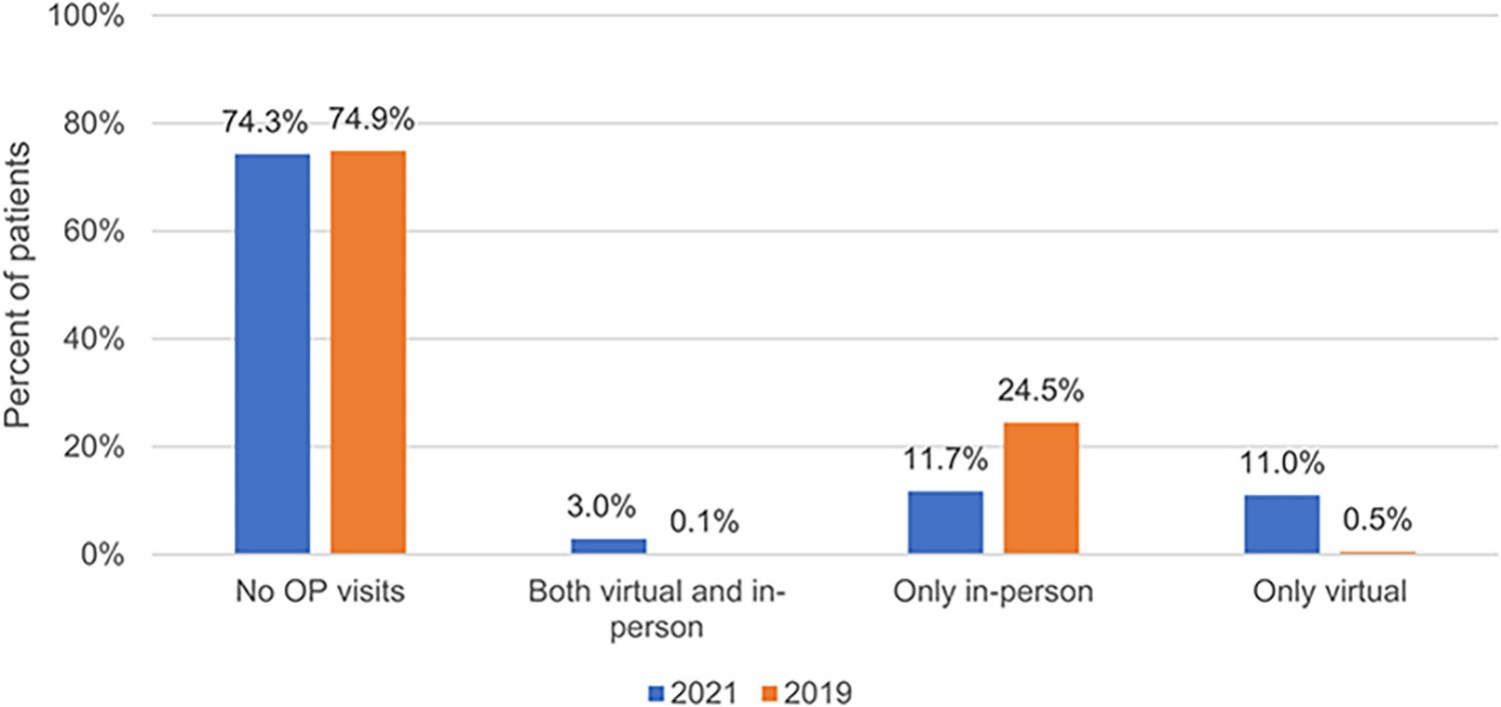
Mode of Outpatient (OP) Visits in the 7 days prior to ED admission between July 1 and Sep 30 in 2019 (n = 1,113,230) and 2021 (n = 1,080,334).

Between July 1, 2021 and September 30, 2021, there were 1,080,334 ED admissions in Ontario and 74% of these admissions had no prior outpatient visits (virtual or in-person) within 7 days of admission (Fig 1). Furthermore, 14% of patients admitted to ED had at least one virtual visit within the 7 days prior to ED admission, occurring on average 2.25 (SD = 2.31) days before the ED admission. Among those who had a virtual or an in-person visit within 7 days of ED admission, 34% had a virtual visit on the same day as the ED admission. (Table 1) In comparison, between July 1, 2019 and September 30, 2019, there were 1,113,230 ED admissions and 75% of admissions had no prior outpatient visits (virtual or in-person) within 7 days of admission and only 0.7% of admissions had a virtual visit 7 days prior to ED admission. (Table 1) Therefore, total ED admissions did not change significantly between 2019 and 2021, however, the availability of virtual visits was much higher in 2021 and as a result a larger percentage of patients who had outpatient visits ahead of ED admission used virtual care (14% of admitted patients in 2021 vs. 0.7% of patients in 2019).

**Table 1:** Sociodemographic characteristics of patients with ED admissions in July 1, 2021- September 30, 2021 vs July 1, 2021- September 30, 2019.

	All patients Jul 1-Sep 20, 2021	All patients Jul 1-Sep 20, 2019	No prior virtual visits Jul 1-Sep 20, 2021	No prior virtual visits Jul 1-Sep 20, 2019	No prior visits (virtual or in-person) Jul 1-Sep 20, 2021	No prior visits (virtual or in-person) Jul 1-Sep 20, 2019	Virtual Visits within 7 days Jul 1-Sep 20, 2021	Virtual Visits within 7 days Jul 1-Sep 20, 2019	In-person Visits only within 7 days Jul 1-Sep 20, 2021	In-person Visits only within 7 days Jul 1-Sep 20, 2019
Number of patients per group and % out of total ED admissions	N = 1,080,334	N = 1,113,230	N = 929,179 (86.01%)	N = 1,105,823 (99.33%)	N = 802,433 (74.28%)	N = 833,255 (74.85%)	N = 151,155 (13.99%)	N = 7,407 (0.67%)	N = 126,746 (11.73%)	N = 272,568 (24.48%)
**Age, N (%)**										
<18	180,889	195,715	162,735	195,142	144,582	155,250	18,154	573	18,153	39,892
	(16.74%)	(17.58%)	(17.51%)	(17.65%)	(18.02%)	(18.63%)	(12.01%)	(7.74%)	(14.32%)	(14.64%)
18–34	241,155	248,772	210,946	246,597	187,122	194,330	30,209	2,175	23,824	52,267
	(22.32%)	(22.35%)	(22.7%)	(22.3%)	(23.32%)	(23.32%)	(19.99%)	(29.36%)	(18.8%)	(19.18%)
35–49	185,769	189,026	158,539	187,167	139,117	143,137	27,230	1,859	19,422	44,030
	(17.2%)	(16.98%)	(17.06%)	(16.93%)	(17.34%)	(17.18%)	(18.01%)	(25.1%)	(15.32%)	(16.15%)
50–64	204,831	213,850	174,337	212,375	150,059	158,197	30,494	1,475	24,278	54,178
	(18.96%)	(19.21%)	(18.76%)	(19.21%)	(18.7%)	(18.99%)	(20.17%)	(19.91%)	(19.15%)	(19.88%)
65+	267,690	265,867	222,622	264,542	181,553	182,341	45,068	1,325	41,069	82,201
	(24.78%)	(23.88%)	(23.96%)	(23.92%)	(22.63%)	(21.88%)	(29.82%)	(17.89%)	(32.4%)	(30.16%)
**Sex, N (%)**										
Female	563,375	582,163	474,322	578,305	404,643	424,729	89,053	3,858	69,679	153,576
	(52.15%)	(52.29%)	(51.05%)	(52.3%)	(50.43%)	(50.97%)	(58.92%)	(52.09%)	(54.98%)	(56.34%)
Male	516,959	531,067	454,857	527,518	397,790	408,526	62,102	3,549	57,067	118,992
	(47.85%)	(47.71%)	(48.95%)	(47.7%)	(49.57%)	(49.03%)	(41.08%)	(47.91%)	(45.02%)	(43.66%)
**Region, N (%)**										
Central	304,291	314,480	246,835	313,156	207,806	216,889	57,456	1,324	39,029	96,267
	(28.17%)	(28.25%)	(26.56%)	(28.32%)	(25.9%)	(26.03%)	(38.01%)	(17.87%)	(30.79%)	(35.32%)
Central East	275,906	277,966	239,363	275,813	209,307	211,043	36,543	2,153	30,056	64,770
	(25.54%)	(24.97%)	(25.76%)	(24.94%)	(26.08%)	(25.33%)	(24.18%)	(29.07%)	(23.71%)	(23.76%)
North	93,309	103,609	87,084	102,386	79,109	88,305	6,225	1,223	7,975	14,081
	(8.64%)	(9.31%)	(9.37%)	(9.26%)	(9.86%)	(10.6%)	(4.12%)	(16.51%)	(6.29%)	(5.17%)
Toronto Central	77,225	80,876	62,678	80,567	52,603	55,592	14,547	309	10,075	24,975
	(7.15%)	(7.26%)	(6.75%)	(7.29%)	(6.56%)	(6.67%)	(9.62%)	(4.17%)	(7.95%)	(9.16%)
West	329,603	336,299	293,219	333,901	253,608	261,426	36,384	2,398	39,611	72,475
	(30.51%)	(30.21%)	(31.56%)	(30.19%)	(31.6%)	(31.37%)	(24.07%)	(32.37%)	(31.25%)	(26.59%)
**Residence, N (%)**										
Rural	120,461	130,792	112,309	129,847	103,599	113,488	8,152	945	8,710	16,359
	(11.15%)	(11.75%)	(12.09%)	(11.74%)	(12.91%)	(13.62%)	(5.39%)	(12.76%)	(6.87%)	(6%)
Urban	939,612	960,154	797,861	953,944	681,515	700,633	141,751	6,210	116,346	253,311
	(86.97%)	(86.25%)	(85.87%)	(86.27%)	(84.93%)	(84.08%)	(93.78%)	(83.84%)	(91.79%)	(92.93%)
Missing	20,261	22,284	19,009	22,032	17,319	19,134	1,252	252	1,690	2,898
	(1.88%)	(2%)	(2.05%)	(1.99%)	(2.16%)	(2.3%)	(0.83%)	(3.4%)	(1.33%)	(1.06%)
**Neighbourhood income quintile, N (%)**										
1 (lowest)	245,567	260,878	213,754	258,723	184,360	196,750	31,813	2,155	29,394	61,973
	(22.73%)	(23.43%)	(23%)	(23.4%)	(22.98%)	(23.61%)	(21.05%)	(29.09%)	(23.19%)	(22.74%)
2	219,408	229,374	188,441	227,808	162,383	171,634	30,967	1,566	26,058	56,174
	(20.31%)	(20.6%)	(20.28%)	(20.6%)	(20.24%)	(20.6%)	(20.49%)	(21.14%)	(20.56%)	(20.61%)
3	214,567	222,006	184,130	220,722	158,999	165,857	30,437	1,284	25,131	54,865
	(19.86%)	(19.94%)	(19.82%)	(19.96%)	(19.81%)	(19.9%)	(20.14%)	(17.33%)	(19.83%)	(20.13%)
4	207,048	206,948	177,256	205,631	153,432	154,243	29,792	1,317	23,824	51,388
	(19.17%)	(18.59%)	(19.08%)	(18.6%)	(19.12%)	(18.51%)	(19.71%)	(17.78%)	(18.8%)	(18.85%)
5 (highest)	189,830	190,182	162,155	189,128	140,370	141,931	27,675	1,054	21,785	47,197
	(17.57%)	(17.08%)	(17.45%)	(17.1%)	(17.49%)	(17.03%)	(18.31%)	(14.23%)	(17.19%)	(17.32%)
Missing	3,914	3,842	3,443	3,811	2,889	2,840	471	31	554	971
	(0.36%)	(0.35%)	(0.37%)	(0.34%)	(0.36%)	(0.34%)	(0.31%)	(0.42%)	(0.44%)	(0.36%)
**Marginalization**										
**Dependency**	2.95 ± 1.48	2.97 ± 1.48	2.98 ± 1.48	2.97 ± 1.48	2.99 ± 1.48	3.02 ± 1.48	2.77 ± 1.48	3.26 ± 1.44	2.91 ± 1.49	2.83 ± 1.48
**Material deprivation**	3.04 ± 1.43	3.09 ± 1.43	3.06 ± 1.43	3.09 ± 1.43	3.06 ± 1.43	3.11 ± 1.43	2.94 ± 1.44	3.35 ± 1.40	3.04 ± 1.44	3.02 ± 1.44
**Residential instability**	3.13 ± 1.43	3.14 ± 1.43	3.13 ± 1.42	3.14 ± 1.43	3.13 ± 1.42	3.14 ± 1.41	3.09 ± 1.49	3.37 ± 1.35	3.16 ± 1.46	3.13 ± 1.48
**Ethnic concentration**	3.05 ± 1.47	3.04 ± 1.47	2.99 ± 1.46	3.05 ± 1.47	2.94 ± 1.46	2.93 ± 1.47	3.45 ± 1.41	2.62 ± 1.35	3.26 ± 1.44	3.40 ± 1.43

### Sociodemographic characteristics

Table 1 lists sociodemographic characteristics of all patients with an ED admission in 2021 and 2019 (Table 1 and also see S2 Appendix for some groups not shown in the manuscript)) across groups of patients with varying use of outpatient care prior to admission. When comparing the periods in 2021 vs. 2019, there were few differences in age, sex, region, rurality of residence, and neighborhood income between patients with no prior visits and patients with only in-person visits prior to their ED admission (<1% changes across). There were shifts in the characteristics of patients who had virtual visits within the 7 days prior to ED admission, with greater proportion of people under 18 (12% in 2021 vs. 8% in 2019) and over 65 (30% in 2021 vs 18% in 2019) using virtual visits, and greater proportion of women having virtual visits in 2021 (59% of users were women in 2021 vs 52% in 2019). A higher proportion of patients with virtual visits prior to ED admission were living in urban regions, especially in Central Ontario (38% of virtual users were in Central Ontario in 2021 vs. 18% in 2019), while those from rural Ontario regions and more specifically North and West regions made up a lower percentage of the virtual users in 2021 relative to 2019, despite absolute numbers increasing (rural: 5% in 2021 vs 13% in 2019; West 24% in 2021 vs. 32% in 2019 and North 4% in 2021 vs. 17% in 2019). Finally, There was a lower proportion of patients living in the lowest neighborhood income regions among those with virtual care use prior to ED admission in 2021 (21% of users were in the lowest quintile vs. 29% in 2019), while the proportion of patients from the highest income regions increased (18% in 2021 vs. 14% in 2019).

### Prior healthcare utilization

The average number of outpatient visits in the year prior to their ED admission across all patients admitted to ED in 2021 and in 2019 was similar (approximately 12 visits, Table 2 and also see S2 Appendix for some groups not shown in the manuscript). Patients with virtual visits prior to ED admission also had similar prior outpatient use in 2021 and 2019, but had more total outpatient visits relative to the entire ED user population (19 visits in 2021 vs. 21 visits in 2019). Across all patients with ED admissions, the average number of ED admissions and hospitalizations in the year prior to their current ED admission were the same in 2021 and 2019 (Mean = 2.5, SD = 3.6 for ED admissions, Mean = 1.5, SD = 1.2 for hospitalizations in 2021, Mean = 2.5, SD = 3.6 for ED admissions, Mean = 1.5, SD = 1.1 for hospitalizations in 2019, Table 2). Patients who used virtual visits prior to ED admission also had similar ED admissions and hospitalizations in 2021 and 2019 (Mean = 2.7, SD = 3.8 ED admissions and Mean = 1.6, SD = 1.2 for hospitalizations in the year prior to admission in 2021 and Mean = 2.7, SD = 3.8 ED admissions and Mean = 1.6, SD = 1.3 for hospitalizations in the year prior to admission 2019).

**Table 2:** Health characteristics of patients with ED admissions in July 1, 2019- September 30, 2019 vs July 1, 2021- September 30, 2019.

	All patients Jul 1-Sep 20, 2021	All patients Jul 1-Sep 20, 2019	No prior virtual visits Jul 1-Sep 20, 2021	No prior virtual visits Jul 1-Sep 20, 2019	No prior visits (virtual or in-person) Jul 1-Sep 20, 2021	No prior visits (virtual or in-person) Jul 1-Sep 20, 2019	Virtual Visits within 7 days Jul 1-Sep 20, 2021	Virtual Visits within 7 days Jul 1-Sep 20, 2019	In-person Visits only within 7 days Jul 1-Sep 20, 2021	In-person Visits only within 7 days Jul 1-Sep 20, 2019
Number of patients per group and % out of total ED admissions	N = 1,080,334	N = 1,113,230	N = 929,179 (86.01%)	N = 1,105,823 (99.33%)	N = 802,433 (74.28%)	N = 833,255 (74.85%)	N = 151,155 (13.99%)	N = 7,407 (0.67%)	N = 126,746 (11.73%)	N = 272,568 (24.48%)
**Asthma**	203,600	222,572	171,869	220,756	147,570	162,633	31,731	1,816	24,299	58,123
**N (%)**	(18.85%)	(19.99%)	(18.5%)	(19.96%)	(18.39%)	(19.52%)	(20.99%)	(24.52%)	(19.17%)	(21.32%)
**CHF**	37,196	51,895	30,305	51,631	23,964	34,088	6,891	264	6,341	17,543
**N (%)**	(3.44%)	(4.66%)	(3.26%)	(4.67%)	(2.99%)	(4.09%)	(4.56%)	(3.56%)	(5%)	(6.44%)
**COPD**	44,642	55,734	36,727	55,247	30,029	37,937	7,915	487	6,698	17,310
**N (%)**	(4.13%)	(5.01%)	(3.95%)	(5%)	(3.74%)	(4.55%)	(5.24%)	(6.57%)	(5.28%)	(6.35%)
**Dementia**	22,035	34,090	18,700	33,978	14,551	23,174	3,335	112	4,149	10,804
**N (%)**	(2.04%)	(3.06%)	(2.01%)	(3.07%)	(1.81%)	(2.78%)	(2.21%)	(1.51%)	(3.27%)	(3.96%)
**HIV**	2,403	2,474	1,996	2,439	1,649	1,647	407	35	347	792
**N (%)**	(0.22%)	(0.22%)	(0.21%)	(0.22%)	(0.21%)	(0.2%)	(0.27%)	(0.47%)	(0.27%)	(0.29%)
**Hypertension**	299,654	327,408	247,745	325,435	203,248	224,901	51,909	1,973	44,497	100,534
**N (%)**	(27.74%)	(29.41%)	(26.66%)	(29.43%)	(25.33%)	(26.99%)	(34.34%)	(26.64%)	(35.11%)	(36.88%)
**Crohn’s**	10,369	11,413	8,344	11,327	6,987	7,766	2,025	86	1,357	3,561
**N (%)**	(0.96%)	(1.03%)	(0.9%)	(1.02%)	(0.87%)	(0.93%)	(1.34%)	(1.16%)	(1.07%)	(1.31%)
**Diabetes**	162,984	169,310	132,973	168,211	108,000	114,459	30,011	1,099	24,973	53,752
**N (%)**	(15.09%)	(15.21%)	(14.31%)	(15.21%)	(13.46%)	(13.74%)	(19.85%)	(14.84%)	(19.7%)	(19.72%)
**Arthritis**	15,908	17,520	12,762	17,382	10,233	11,507	3,146	138	2,529	5,875
**N (%)**	(1.47%)	(1.57%)	(1.37%)	(1.57%)	(1.28%)	(1.38%)	(2.08%)	(1.86%)	(2%)	(2.16%)
**ED visits in past 365d**,	2.46 ± 3.62	2.53 ± 3.57	2.43 ± 3.59	2.52 ± 3.56	2.38 ± 3.42	2.47 ± 3.46	2.65 ± 3.78	3.37 ± 4.44	2.69 ± 4.42	2.68 ± 3.85
Mean ± SD										
**Hospitalizations in past 365d** Mean ± SD	1.48 ± 1.15	1.50 ± 1.14	1.47 ± 1.14	1.50 ± 1.14	1.45 ± 1.12	1.48 ± 1.11	1.55 ± 1.18	1.63 ± 1.28	1.52 ± 1.19	1.53 ± 1.18
**Physician visits in past 365d** Mean ± SD	12.56 ± 18.24	11.68 ± 16.84	11.38 ± 16.91	11.62 ± 16.75	10.30 ± 15.24	9.96 ± 14.48	19.04 ± 23.26	20.70 ± 24.44	17.54 ± 23.41	16.33 ± 21.25
**Number of outpatient visits in past 7d**, Mean ± SD	1.32 ± 0.65	1.25 ± 0.56	1.17 ± 0.46	1.25 ± 0.56	n/a	n/a	1.45 ± 0.76	1.34 ± 0.68	1.17 ± 0.46	1.25 ± 0.56
**Number of days between virtual/in-person and ED visits**,	2.25 ± 2.31	2.49 ± 2.34	n/a	n/a	n/a	n/a	2.25 ± 2.31	2.49 ± 2.34	2.55 ± 2.47	2.80 ± 2.46
Mean ± SD										
**Virtual/in-person visit same day as ED visit,**	51,448	2,211	0	0	0	0	51,448	2,211	42,845	76,054
	(4.76%)	(0.2%)	(0.00%)	(0.00%)	(0.00%)	(0.00%)	(34.04%)	(29.85%)	(33.8%)	(27.9%)
**N (%)**										
**ED visit resulted in hospitalization, N (%)**	98,735	94,415	79,860	93,747	61,462	59,781	18,875	668	18,398	33,966
	(9.14%)	(8.48%)	(8.59%)	(8.48%)	(7.66%)	(7.17%)	(12.49%)	(9.02%)	(14.52%)	(12.46%)

**Fig 2:**
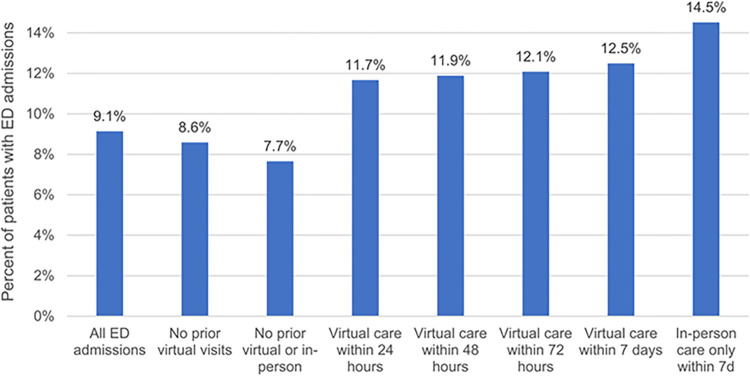
Percentage of patients with ED admissions that resulted in hospitalization across subgroups of patients with varying use of virtual care prior to admission.

When examining all patients with ED admissions, the average number of outpatient visits per patient in the 7 days prior to admission did not change (Mean = 1.3, SD = 0.7 in both 2021 and 2019). Patients with prior virtual visits were more likely to be hospitalized after their ED admission (13%) than patients with no prior outpatient care (7.7%), but patients with in-person visits prior to ED admissions were even more likely to be hospitalized (14.5%) (Fig 2).

### Reason for ED admission

The 5 most common reasons for ED admission in July 1, 2021- September 30, 2021 were chest pain (14%), abdominal pain (11%), urinary tract infection (9%), acute upper respiratory infection (6%) and open wound of finger(s) (5%) across all ED admission. These reasons were consistent across patient groups with little variation on volumes of these admissions (Table 3). The most common reasons for the last virtual visit prior to ED admission were “other ill-defined conditions” (12%), gastrointestinal issues (12%), anxiety (11%), chest pain (8%) and leg cramps (7%). The same reasons were seen in the in-person visits only group with the exception that gastrointestinal and anxiety issues were the top two reasons for an in-person visit prior to ED admission (13%) ahead of “other ill-defined conditions” (Table 4).

**Table 3:** Top 5 Reasons for ED admission, N (%), July 1, 2021- September 30, 2021.

	All patients	No prior virtual visits	No prior visits (virtual or in-person)	Virtual Visits within 7 days	In-person Visits only within 7 days
	N = 1,080,334	N = 929,179	N = 802,433	N = 151,155	N = 126,746
Chest pain N (%)	38,183 (14.1%)	31,859 (13.8%)	27,735 (13.6%)	6,324 (15.8%)	4,124 (15.1%)
Abdominal pain N (%)	29,859 (11.0%)	24,688 (10.7%)	21,029 (10.3%)	5,171 (12.9%)	3,659 (13.4%)
Urinary tract infection N (%)	25,309 (9.3%)	21,648 (9.3%)	18,981 (9.3%)	3,661 (9.1%)	2,667 (9.8%)
Acute upper respiratory infection N (%)	15,235 (5.6%)	13,063 (5.6%)	12,004 (5.9%)	2,172 (5.4%)	1,059 (3.9%)
Open wound of finger(s) N (%)	14,781	14,035	13,181	746	854
	(5.4%)	(6.1%)	(6.5%)	(1.9%)	(3.1%)

**Table 4:** Top 5 reasons for the last virtual visit prior to ED admission, N (%), July 1, 2021- September 30, 2021.

	All patients	Virtual Visits within 7 days	In-person Visits only within 7 days
	N = 1,080,334	N = 151,155	N = 126,746
Other ill-defined conditions N (%)	8,926 (11.6%)	8,926 (11.6%)	4,601 (9.43%)
Gastrointestinal issues N (%)	8,880 (11.6%)	8,880 (11.6%)	6,522 (13.36%)
Anxiety N (%)	8,263 (10.8%)	8,263 (10.8%)	6,285 (12.88%)
Chest pain N (%)	5,787 (7.5%)	5,787 (7.5%)	4,008 (8.21%)
Leg cramps N (%)	5,178 (6.8%)	5,178 (6.8%)	3,723 (7.63%)

### Physician characteristics

There were no differences in physician characteristics (age, sex, years in practice, patient volume, and region of practice) between those who provided a virtual visit right before admission versus those who provided either a virtual or in-person visit in July 1, 2021- September 30, 2021. (Table 5)

**Table 5:** Comparison of physicians who provided the last outpatient visit before ED admissions across care modalities (virtual vs. virtual or in-person) in July 1, 2021- September 30, 2021, Mean ± SD.

		All physicians	Primary Care physicians only	Psychiatry	Pediatrics	Internal Medicine	Obstetrics & Gynaecology
		N = 19,704	N = 11,189	N = 1,405	N = 781	N = 763	N = 567
Age Mean (SD)							
Virtual Visit	Mean (SD)	49.39 ± 12.51	49.09 ± 12.91	54.05 ± 13.40	50.53 ± 12.67	49.84 ± 13.81	49.31 ± 11.29
Virtual or In-person visit	Mean (SD)	49.33 ± 12.67	48.95 ± 13.00	53.56 ± 13.48	49.87 ± 12.48	48.64 ± 14.02	49.89 ± 11.61
Sex, N (%)							
Virtual Visit	Female	8,920	5,611	615	465	226 (29.6%)	375 (66.1%)
		(45.3%)	(50.2%)	(43.8%)	(59.5%)		
	Male	10,784	5,578	790	316	537 (70.4%)	192 (33.9%)
		(54.7%)	(49.9%)	(56.2%)	(40.5%)		
Virtual or In-person visit	Female	11,096	6,335	748	680	364 (32.2%)	550 (64.6%)
		(42.9%)	(48.3%)	(44.3%)	(59.8%)		
	Male	14,791	6,777	942	458	768 (67.8%)	302 (35.5%)
		(57.1%)	(51.7%)	(55.7%)	(40.3%)		
Region of practice, N (%)							
Virtual Visit	Missing	122	80	11	7	*1–5	*1–5
		(0.6%)	(0.7%)	(0.8%)	(0.9%)		
	Central	5,952	3,574	283	272	220	159
		(30.2%)	(31.9%)	(20.1%)	(34.8%)	(28.8%)	(28.0%)
	East	4,667	2,705	278	176	166	138
		(23.7%)	(24.2%)	(19.8%)	(22.5%)	(21.8%)	(24.3%)
	North	951	605	47	11	*36–40	*23–27
		(4.8%)	(5.4%)	(3.4%)	(1.4%)		
	Toronto	3,049	1,446	471	141	86	103
		(15.5%)	(12.9%)	(33.5%)	(18.1%)	(11.3%)	(18.2%)
	West	4,963	2,779	315	174	250	139
		(25.2%)	(24.8%)	(22.4%)	(22.3%)	(32.8%)	(24.5%)
Virtual or In-person visit	Missing	181	106	16	10	8	3
		(0.7%)	(0.8%)	(1.0%)	(0.9%)	(0.7%)	(0.4%)
	Central	7,454	3,986	335	381	327	225
		(28.8%)	(30.4%)	(19.8%)	(33.5%)	(28.9%)	(26.4%)
	East	6,268	3,220	349	253	244	224
		(24.2%)	(24.6%)	(20.7%)	(22.2%)	(21.6%)	(26.3%)
	North	1,406	846	68	32	51	38
		(5.4%)	(6.5%)	(4.0%)	(2.8%)	(4.5%)	(4.5%)
	Toronto	3,917	1,632	547	180	140	146
		(15.1%)	(12.5%)	(32.4%)	(15.8%)	(12.4%)	(17.1%)
	West	6,661	3,322	375	282	362	216
		(25.7%)	(25.3%)	(22.2%)	(24.8%)	(32.0%)	(25.4%)
Years in practice, mean (SD)							
Virtual Visit	Mean (SD)	22.54 ± 13.71	21.96 ± 14.08	27.20 ± 14.99	24.28 ± 14.06	22.57 ± 15.72	22.68 ± 12.35
Virtual or In-person visit	Mean (SD)	22.41 ± 13.78	21.75 ± 14.14	26.60 ± 15.09	23.45 ± 13.88	21.32 ± 15.92	23.29 ± 12.92
Patient volume per day during observation window, mean (SD)							
Virtual Visit	Mean (SD)	16.15 ± 11.38	18.63 ± 12.43	6.86 ± 4.79	13.69 ± 10.01	12.16 ± 8.73	18.65 ± 9.27
Virtual or In-person visit	Mean (SD)	14.88 ± 11.56	17.36 ± 12.64	6.52 ± 4.79	11.61 ± 9.39	10.51 ± 8.43	18.06 ± 8.83

### Interpretation

We found no rise in ED admission volumes and pre-admission outpatient care in the summer of 2021 relative to 2019. For patients with outpatient care in the 7 days prior to admission (25% of all patients), the modality of outpatient care shifted from patients having almost exclusively in-person visits in 2019 to patients having either virtual visits only or in-person care only in the week prior to their ED admission. Very few patients (3%) had a mix of both virtual and in-person care in the 7 days before their admission. Patients who had virtual visits prior to ED admission had more outpatient visits, but similar ED and hospitalization rates in the year prior to admission. They were also more likely to be hospitalized after their ED admission than patients with no prior visits. The most common reasons for ED admission were similar across groups of patients with varying levels of virtual care use prior to ED admission.

Early in the pandemic, there were numerous reports of a decline in ED admissions across the globe [12–14]. However, stable pandemic periods, such as the one we report on in this study, have shown a return to regular ED use [14–16]. Consistent with these findings, ED admission volumes in Ontario remained nearly identical to those during a matched pre-pandemic period and the use of outpatient services prior to ED admissions did not increase. Therefore, the rise in use of virtual outpatient care in Ontario (about 50% of all outpatient care [17]) did not appear to lead to increased use of the ED. Furthermore, patients were admitted to the ED for similar reasons in both 2021 and 2019 with no evident shift in the top causes of ED admissions. Of note is that a greater proportion of those admitted to the ED were hospitalised in 2021 (9.14%) compared to 2019 (8.14%), which may suggest patients were more severely ill on ED admission. To our knowledge, this is the first study to look at the relationship between virtual visits use and ED use during the pandemic.

Patients who went to the ED with prior virtual visits were more likely to be hospitalized than those without any prior outpatient care (despite similar ED and hospital utilization in the year prior). This suggests that virtual visits were sufficient for physicians to advise patients to go to the emergency and an in-person outpatient visit was not required (only 3% of patients had a mix of virtual and in-person visits in the week prior to ED admission). Our finding is supported by the success of numerous emergency department clinics that introduced virtual visits during the pandemic [18–20].

Virtual visits were more prevalent in urban and higher income regions. Patients who went into the ED with a prior virtual visit in the week leading to their admission generally seemed to have better access to outpatient care, as evidenced by a higher number of outpatient visits in the year before their ED admission. While this may suggest greater severity of disease, there were no differences in the number of hospitalizations or ED admissions in the year prior to admission suggesting that the difference may lie in better access to outpatient care. This supports a growing concern that the shift towards virtual visits may be further limiting marginalized patient populations’ access to care [21]. This reinforces the notion that health equity considerations should always be at the forefront of the implementation of virtual care programs [22, 23].

As virtual visits continue to be an integral part of medicine, it is reassuring to see that despite its high use during the COVID-19 pandemic, increases in ED use were not observed. For this study we examined patients who were visiting the ED, but what remains to be examined is how virtual visits affect downstream use of not only the ED, but also other types of outpatient care and diagnostics. The balance of virtual and in-person care may shift over time, so it is important to examine the relationship between virtual outpatient use and ED use and explore how these relationships change as practices build virtual visits into their workflows and better understand its value. Virtual visits may have differential impact on various ED admission diagnoses, which can be the subject of future work. It is noteworthy that despite the ease of virtual visits, similarly large proportions of patients go straight to the ED without seeking outpatient care before and during COVID, likely due to poor virtual visit access. This supports the idea that we need to make the type of care available in ED or holistic care more accessible overall so that more low acuity care shifts outside the emergency department [24].

### Limitations

Limitations of this study include the use of administrative databases which lack the clinical granularity to assess details such as the appropriateness of the visit(s). Second, the recent temporary COVID-19 virtual billing codes do not distinguish between telephone and video, and therefore we were unable to make comparisons of the various modalities of virtual visits. Lastly, the findings in this report are descriptive only and span a short period of three months, and therefore results are only preliminary and may not be generalizable in different contexts.

## Conclusions

Despite concerns that access to virtual visits may lead to a rise in ED admissions or a greater use of outpatient services prior to ED admissions (in the form of patients having both virtual and in-person visits), the net amount of ED admissions and outpatient care prior to admission remained the same over a period of the COVID-19 pandemic when cases were relatively stable. Virtual visits seem to be able to appropriately triage patients to the ED and may even prove beneficial for diverting patients away from the ED when an ED admission is not appropriate.

## Supporting information

S1 Appendix Methods details. (DOCX)

S2 Appendix Results details. (DOCX)
