# Supplementary material for: Virtual care use prior to emergency department admissions during a stable COVID-19 period in Ontario, Canada
Source: PLoS One. 2023 Apr 28;18(4):e0277065. doi: 10.1371/journal.pone.0277065 (PMC10146565; doi:10.1371/journal.pone.0277065)
Supplement: S1 Appendix — (DOCX) [file pone.0277065.s001.docx]

# Supplement

## Methods

We applied the following exclusion criteria to the cohort: invalid health card number, non-Ontario resident, missing key demographic identifiers (e.g., birth date, sex), and we also excluded those who had another ED admission within 7 days prior to the start of the study window.

| **Exclusions for Cohorts 1a/1b** *(in order)  (common exclusions are listed in grey italics for consideration)* | *Step* | Description |
| --- | --- | --- |
|  | 1 | Invalid ICES Key Number |
|  | 2 | Invalid birth date (i.e., missing or after index date) |
|  | 3 | Invalid death date (i.e., before index date) |
|  | 4 | Missing or invalid sex |
|  | 5 | Age <0 or >105 |
|  | 6 | Non-Ontario resident at index |
|  | 7 | OHIP ineligible on ED visit date +/- 7 days |
|  | 8 | Had another ED visit within 7 days prior to July 1 start date |
|  | 9 | **Please apply exclusion criteria for all cohorts defined above.**  **Please report number excluded for each.** |

**Patient characteristics** (data to be extracted on date of ED visit (i.e., index date)):

- Age (with categories: <18, 18-34, 35-49, 50-64, 65+) [RPDB], n(%)
- Male Sex [RPDB] (n,%)
- Region (based on clustering previously existing LHINs into five Ontario Health Interim and Transitional Regions), n(%):
  - **North:** North West, North East
  - **West:** Erie St. Clair, South West, Waterloo Wellington, Hamilton Niagara Haldimand Brant
  - **Central:** Central West, Central, Mississauga Halton, North Simcoe Muskoka
  - **East:** Central East, South East and Champlain
  - **Toronto**: Toronto Central
- Neighbourhood Income quintile, n(%) [RPDB, PCCF] – based on patient’s postal code
- Ontario Marginalization Index [ONMARG] – mean score for each of the four dimensions (economic, ethno-racial, age-based, social)
- Rurality (using RIO2008 score with categories: urban <40 versus rural ≥40) [RPDB], n(%)
- Major disease diagnosis [use ICES-derived cohorts: ASTHMA, CHF, COPD, DEMENTIA, HIV, HYPER, OCCC, ODD, OMID, ORAD]: list diagnoses, n(%)
- Overall healthcare utilization in the past year [OHIP, NACRS, DAD, SDS]:
  - Number of ED admissions, mean (SD) and median (IQR). *NB: do not include index ED visit.*
  - Number of hospitalizations, mean (SD) and median (IQR)
  - Number of physician visits, mean (SD) and median (IQR). This can include visits captured in the categories above. *NB: do not include visits in the exposure time frame (i.e., 7-d prior). Window of interest here is: indexdate-365 until indexdate-7.*
- Reason for ED visit [NACRS]: list top 20 reasons (DX10CODE1), n(%)
- DXCODE on virtual visit [OHIP]: list top 20 diagnosis codes, n(%)
- Number of outpatient visits in past 7 days [OHIP], mean (SD) or median(IQR)
- Mode of outpatient visits in past 7 days [OHIP]
  - Proportion (n,%) of patients with only virtual visits
  - Proportion (n,%) of patients with only in-person visits
  - Proportion (n,%) of patients with both virtual and in-person visits
- Number of days between virtual visit and ED visit [OHIP, NACRS], mean(SD) or median(IQR)
- For patients with a virtual visit: use of B vs K code on virtual visit [OHIP], n(%)
- Number of ED visits that resulted in hospitalization [NACRS, DAD], n(%)
- Number of ED visits that are Canadian Triage and Acuity Scale (CTAS) level 4 or 5 [NACRS], n(%)
- Number of patients who had their virtual visit on the same day as their ED visit, n (%)
- Rostering status of patient at date of index ED visit, n(%). Use macro %pcprovider, and include patients who are rostered virtually.

Physician Characteristics

**Physician characteristics** (data to be extracted on date of patient’s ED visit):

- Age (as of 01-Jul) [IPDB, CPDB], mean(SD) or median(IQR). If missing Age, use birth year.
- Male sex [IPDB, CPDB], n(%)
- Region of practice (based on clustering previously existing LHINs into five Ontario Health Interim and Transitional Regions), n(%) [IPDB – MAINLHIN (if missing, used LHIN), CPDB]:
  - **North:** North West, North East
  - **West:** Erie St. Clair, South West, Waterloo Wellington, Hamilton Niagara Haldimand Brant
  - **Central:** Central West, Central, Mississauga Halton, North Simcoe Muskoka
  - **East:** Central East, South East and Champlain
  - **Toronto**: Toronto Central
- Years in practice (categorical) [IPDB, CPDB], n(%). Use gradyear variable in IPDB and eligdate in CPDB
- Average patient volume per day during Jun 24 to Sept 30 window, mean(SD). Use total billing days in that time frame as denominator.
