# Supplementary material for: Virtual care use prior to emergency department admissions during a stable COVID-19 period in Ontario, Canada
Source: PLoS One. 2023 Apr 28;18(4):e0277065. doi: 10.1371/journal.pone.0277065 (PMC10146565; doi:10.1371/journal.pone.0277065)
Supplement: S2 Appendix — (DOCX) [file pone.0277065.s002.docx]

Table 1 Sociodemographic characteristics of patients with ED admissions in July 1, 2021- September 30, 2021 vs July 1, 2021- September 30, 2019.

|  | Virtual Visits within 24 hours  Jul 1-Sep 20, 2021 | Virtual Visits within 24 hours  Jul 1-Sep 20, 2019 | Virtual Visits within 48 hours Jul 1-Sep 20, 2021 | Virtual Visits within 48 hours Jul 1-Sep 20, 2019 | Virtual Visits within 72 hours Jul 1-Sep 20, 2021 | Virtual Visits within 72 hours Jul 1-Sep 20, 2019 |
| --- | --- | --- | --- | --- | --- | --- |
|  | **N=76,027** | **N=3,313** | **N=92,749** | **N=4,196** | **N=106,801** | **N=4,974** |
| Age, N (%) |  |  |  |  |  |  |
| <18 | 12,073 (15.88%) | 339 (10.23%) | 13,731 (14.8%) | 421 (10.03%) | 14,900 (13.95%) | 469 (9.43%) |
| 18-34 | 15,378 (20.23%) | 927 (27.98%) | 18,799 (20.27%) | 1,195 (28.48%) | 21,624 (20.25%) | 1,435 (28.85%) |
| 35-49 | 13,423 (17.66%) | 708 (21.37%) | 16,529 (17.82%) | 948 (22.59%) | 19,085 (17.87%) | 1,164 (23.4%) |
| 50-64 | 14,386 (18.92%) | 663 (20.01%) | 17,858 (19.25%) | 824 (19.64%) | 20,891 (19.56%) | 980 (19.7%) |
| 65+ | 20,767 (27.32%) | 676 (20.4%) | 25,832 (27.85%) | 808 (19.26%) | 30,301 (28.37%) | 926 (18.62%) |
| Sex, N (%) |  |  |  |  |  |  |
| Female | 44,354 (58.34%) | 1,740 (52.52%) | 54,326 (58.57%) | 2,194 (52.29%) | 62,631 (58.64%) | 2,593 (52.13%) |
| Male | 31,673 (41.66%) | 1,573 (47.48%) | 38,423 (41.43%) | 2,002 (47.71%) | 44,170 (41.36%) | 2,381 (47.87%) |
| Region, N (%) |  |  |  |  |  |  |
| Central | 30,796 (40.51%) | 593 (17.9%) | 37,046 (39.94%) | 753 (17.95%) | 42,144 (39.46%) | 877 (17.63%) |
| Central East | 17,725 (23.31%) | 1,023 (30.88%) | 21,848 (23.56%) | 1,273 (30.34%) | 25,292 (23.68%) | 1,506 (30.28%) |
| North | 2,483 (3.27%) | 439 (13.25%) | 3,240 (3.49%) | 590 (14.06%) | 3,895 (3.65%) | 724 (14.56%) |
| Toronto Central | 7,657 (10.07%) | 146 (4.41%) | 9,242 (9.96%) | 182 (4.34%) | 10,566 (9.89%) | 216 (4.34%) |
| West | 17,366 (22.84%) | 1,112 (33.56%) | 21,373 (23.04%) | 1,398 (33.32%) | 24,904 (23.32%) | 1,651 (33.19%) |
| Residence, N (%) |  |  |  |  |  |  |
| Rural | 3,298 (4.34%) | 387 (11.68%) | 4,252 (4.58%) | 494 (11.77%) | 5,123 (4.8%) | 598 (12.02%) |
| Urban | 72,175 (94.93%) | 2,834 (85.54%) | 87,787 (94.65%) | 3,582 (85.37%) | 100,849 (94.43%) | 4,227 (84.98%) |
| Missing | 554 (0.73%) | 92 (2.78%) | 710 (0.77%) | 120 (2.86%) | 829 (0.78%) | 149 (3%) |
| Neighbourhood income quintile, N (%) |  |  |  |  |  |  |
| 1 (lowest) | 15,226 (20.03%) | 890 (26.86%) | 18,711 (20.17%) | 1,147 (27.34%) | 21,834 (20.44%) | 1,376 (27.66%) |
| 2 | 15,281 (20.1%) | 691 (20.86%) | 18,752 (20.22%) | 879 (20.95%) | 21,648 (20.27%) | 1,039 (20.89%) |
| 3 | 15,576 (20.49%) | 591 (17.84%) | 19,011 (20.5%) | 752 (17.92%) | 21,804 (20.42%) | 898 (18.05%) |
| 4 | 15,222 (20.02%) | 614 (18.53%) | 18,538 (19.99%) | 761 (18.14%) | 21,258 (19.9%) | 889 (17.87%) |
| 5 (highest) | 14,471 (19.03%) | 508 (15.33%) | 17,428 (18.79%) | 638 (15.2%) | 19,906 (18.64%) | 750 (15.08%) |
| Missing | 251 (0.33%) | 19 (0.57%) | 309 (0.33%) | 19 (0.45%) | 351 (0.33%) | 22 (0.44%) |
| Marginalization |  |  |  |  |  |  |
| Dependency | 2.69 ± 1.47 | 3.23 ± 1.44 | 2.71 ± 1.47 | 3.23 ± 1.44 | 2.73 ± 1.47 | 3.24 ± 1.44 |
| Material deprivation | 2.90 ± 1.43 | 3.26 ± 1.42 | 2.91 ± 1.43 | 3.29 ± 1.42 | 2.92 ± 1.43 | 3.30 ± 1.41 |
| Residential instability | 3.03 ± 1.51 | 3.31 ± 1.36 | 3.04 ± 1.50 | 3.33 ± 1.36 | 3.05 ± 1.50 | 3.33 ± 1.35 |
| Ethnic concentration | 3.55 ± 1.38 | 2.64 ± 1.35 | 3.53 ± 1.39 | 2.64 ± 1.36 | 3.51 ± 1.39 | 2.64 ± 1.35 |

Table 2. Health characteristics of patients with ED admissions in July 1, 2019- September 30, 2019.

|  | Virtual Visits within 24 hours  Jul 1-Sep 20, 2021 | Virtual Visits within 24 hours  Jul 1-Sep 20, 2019 | Virtual Visits within 48 hours Jul 1-Sep 20, 2021 | Virtual Visits within 48 hours Jul 1-Sep 20, 2019 | Virtual Visits within 72 hours Jul 1-Sep 20, 2021 | Virtual Visits within 72 hours Jul 1-Sep 20, 2019 |
| --- | --- | --- | --- | --- | --- | --- |
|  | **N=76,027** | **N=3,313** | **N=92,749** | **N=4,196** | **N=106,801** | **N=4,974** |
| Asthma | 15,224 (20.02%) | 745 (22.49%) | 18,768 (20.24%) | 985 (23.47%) | 21,810 (20.42%) | 1,182 (23.76%) |
| CHF | 3,039 (4%) | 119 (3.59%) | 3,804 (4.1%) | 144 (3.43%) | 4,521 (4.23%) | 167 (3.36%) |
| COPD | 3,384 (4.45%) | 182 (5.49%) | 4,276 (4.61%) | 246 (5.86%) | 5,123 (4.8%) | 292 (5.87%) |
| Dementia | 1,631 (2.15%) | 58 (1.75%) | 1,997 (2.15%) | 72 (1.72%) | 2,316 (2.17%) | 83 (1.67%) |
| HIV | 159 (0.21%) | 17 (0.51%) | 205 (0.22%) | 21 (0.5%) | 259 (0.24%) | 24 (0.48%) |
| Hypertension | 23,581 (31.02%) | 927 (27.98%) | 29,595 (31.91%) | 1,144 (27.26%) | 34,765 (32.55%) | 1,341 (26.96%) |
| Crohn's | 895 (1.18%) | 33 (1%) | 1,139 (1.23%) | 51 (1.22%) | 1,330 (1.25%) | 58 (1.17%) |
| Diabetes | 13,518 (17.78%) | 485 (14.64%) | 16,958 (18.28%) | 596 (14.2%) | 19,976 (18.7%) | 714 (14.35%) |
| Arthritis | 1,373 (1.81%) | 59 (1.78%) | 1,726 (1.86%) | 70 (1.67%) | 2,046 (1.92%) | 89 (1.79%) |
| ED visits in past 365d | 2.40 ± 3.14 | 2.99 ± 3.94 | 2.48 ± 3.44 | 3.11 ± 4.21 | 2.53 ± 3.48 | 3.20 ± 4.36 |
| Hospitalizations in past 365d | 1.47 ± 1.06 | 1.55 ± 1.29 | 1.49 ± 1.07 | 1.57 ± 1.30 | 1.50 ± 1.09 | 1.60 ± 1.27 |
| Physician visits in past 365d | 17.11 ± 22.34 | 16.46 ± 21.73 | 17.68 ± 22.70 | 17.66 ± 23.11 | 18.09 ± 22.89 | 18.48 ± 23.21 |
| Number of outpatient visits in past 7d | 1.52 ± 0.82 | 1.35 ± 0.70 | 1.51 ± 0.81 | 1.35 ± 0.70 | 1.50 ± 0.80 | 1.35 ± 0.70 |
| Number of days between virtual/in-person and ED visits | 0.32 ± 0.47 | 0.33 ± 0.47 | 0.63 ± 0.77 | 0.68 ± 0.80 | 0.94 ± 1.08 | 1.05 ± 1.12 |
| Virtual/in-person visit same day as ED visit, N (%) | 51,448 (67.67%) | 2,211 (66.74%) | 51,448 (55.47%) | 2,211 (52.69%) | 51,448 (48.17%) | 2,211 (44.45%) |
| ED visit resulted in hospitalization, N (%) | 8,874 (11.67%) | 345 (10.41%) | 11,025 (11.89%) | 411 (9.8%) | 12,897 (12.08%) | 466 (9.37%) |

Table 3: Top 5 Reasons for ED admission, N (%), July 1, 2021- September 30, 2021

|  | Virtual Visits within 24 hours | Virtual Visits within 48 hours | Virtual Visits within 72 hours |
| --- | --- | --- | --- |
|  | N=76,027 | N=92,749 | N=106,801 |
| Chest pain | 3,321  (15.7%) | 3,964  (15.6%) | 4,497  (15.5%) |
| Abdominal pain | 2,480  (11.8%) | 3,066  (12.1%) | 3,536  (12.2%) |
| Urinary tract infection | 1,736  (8.2%) | 2,176  (8.6%) | 2,591  (9.0%) |
| Acute upper respiratory infection | 1,429  (6.8%) | 1,646  (6.5%) | 1,797  (6.2%) |
| Open wound of finger(s) | 272  (1.3%) | 345  (1.4%) | 439  (1.5%) |

Table 4: Top 5 reasons for the last virtual visit prior to ED admission, N (%), July 1, 2021- September 30, 2021

|  | Virtual Visits within 24 hours | Virtual Visits within 48 hours | Virtual Visits within 72 hours |
| --- | --- | --- | --- |
|  | **N=76,027** | **N=92,749** | **N=106,801** |
| Other ill-defined conditions | 4,961 (12.2%) | 5,935 (12.1%) | 6,635 (11.9%) |
| Gastrointestinal issues | 5,634 (13.9%) | 6,444 (13.1%) | 7,060 (12.6%) |
| Anxiety | 2,921 (7.2%) | 3,968 (8.1%) | 4,941 (8.8%) |
| Chest pain | 4,086 (10.1%) | 4,506 (9.2%) | 4,832 (8.7%) |
| Leg cramps | 2,755 (6.8%) | 3,313 (6.8%) | 3,774 (6.8%) |
